# No Geographic Correlation between Lyme Disease and Death Due to 4 Neurodegenerative Disorders, United States, 2001–2010

**DOI:** 10.3201/eid2111.150778

**Published:** 2015-11

**Authors:** Joseph D. Forrester, Kiersten J. Kugeler, Anna E. Perea, Daniel M. Pastula, Paul S. Mead

**Affiliations:** Centers for Disease Control and Prevention, Fort Collins, Colorado, USA (J.D. Forrester, K.J. Kugeler, A.E. Perea, D.M. Pastula, P.S. Mead);; Centers for Disease Control and Prevention, Atlanta, Georgia, USA (J.D. Forrester, D.M. Pastula)

**Keywords:** Lyme disease, Alzheimer disease, Parkinson disease, amyotrophic lateral sclerosis, ALS, multiple sclerosis, multiple sclerosis, MS, geographic distribution, neurodegenerative diseases, neurodegenerative disorders, vectorborne diseases, vector-borne infections, zoonoses, United States, Borrelia burgdorferi, bacteria

## Abstract

Associations between Lyme disease and certain neurodegenerative diseases have been proposed, but supportive evidence for an association is lacking. Similar geographic distributions would be expected if 2 conditions were etiologically linked. Thus, we compared the distribution of Lyme disease cases in the United States with the distributions of deaths due to Alzheimer disease, amyotrophic lateral sclerosis (ALS), multiple sclerosis (MS), and Parkinson disease; no geographic correlations were identified. Lyme disease incidence per US state was not correlated with rates of death due to ALS, MS, or Parkinson disease; however, an inverse correlation was detected between Lyme disease and Alzheimer disease. The absence of a positive correlation between the geographic distribution of Lyme disease and the distribution of deaths due to Alzheimer disease, ALS, MS, and Parkinson disease provides further evidence that Lyme disease is not associated with the development of these neurodegenerative conditions.

## Abstract 2

Death rates for these disorders were not associated with incidence of confirmed Lyme disease cases.

Lyme disease is a complex, multisystem tickborne illness caused by the spirochete *Borrelia burgdorferi* (*1*). Each year in the United States, >30,000 cases are reported, but the actual number of infections may be 10-fold higher (*1*,*2*). Lyme disease cases are most commonly reported from the Northeast, mid-Atlantic, and upper Midwest regions of the United States (*3*). Central nervous system infection resulting in early neurologic Lyme disease, and more rarely late neurologic Lyme disease, is well documented (*4*,*5*). Because of the neurotropism of Lyme disease, speculative websites and articles and even peer-reviewed journals have purported causal associations between Lyme disease and several neurodegenerative disorders, including Alzheimer disease, amyotrophic lateral sclerosis (ALS), multiple sclerosis (MS), and Parkinson disease (*6*–*11*). Researchers have critically evaluated these proposed biologic associations between Lyme disease and Alzheimer disease, ALS, MS, and Parkinson disease, but none have found evidence of an association (*12*–*21*). We hypothesized that, if there is a link between *B. burgdorferi* infection and subsequent development of Alzheimer disease, ALS, MS, or Parkinson disease, the geographic distribution of these neurodegenerative disorders should correlate with that of Lyme disease. To determine if such a correlation exists, we compared the distribution of confirmed cases of Lyme disease in the United States with the distribution of deaths due to these 4 neurodegenerative disorders.

## Methods

We compared Lyme disease incidence rates in each state with death rates for Alzheimer disease, ALS, MS, and Parkinson disease. Reports of confirmed Lyme disease cases submitted to the National Notifiable Diseases Surveillance System during 2001–2010 (*2*) were used to calculate state-specific, age-adjusted incidence rates of Lyme disease. Age-adjusted death rates of Alzheimer disease, ALS, MS, and Parkinson disease during the same time period were obtained from the CDC WONDER (Centers for Disease Control and Prevention Wide-ranging Online Data for Epidemiologic Research) database (http://wonder.cdc.gov/WelcomeT.html). Codes for underlying cause of death from the International Classification of Diseases, Tenth Revision, Clinical Modification (http://www.cdc.gov/nchs/icd/icd10cm.htm), were as follows: G30, Alzheimer disease; G12.2, motor neuron disease (ALS); G35, MS; and G20, Parkinson disease. We standardized the Lyme disease incidence rates and neurodegenerative disease death rates to the 2000 US population by using 10-year age groups (http://www.census.gov/2000census/data/).

We used the Moran’s I test for spatial autocorrelation to assess geographic clustering of state incidence rates of Lyme disease and of death rates for the 4 neurologic disorders by using ArcGIS 10.1 (ESRI, Redlands, CA, USA). Geographic correlation between Lyme disease incidence and death rates for each of the other conditions was assessed by using the Spearman rank correlation (*r_s_*) to compare pairwise state rates. A subanalysis comparing pairwise state rates by sex was similarly performed. Rates for male patients who died of MS in Hawaii and the District of Columbia were not included in the analysis because of CDC WONDER data use restrictions. Analyses were conducted by using SAS version 9.3 (SAS Institute, Inc., Cary, NC, USA). Because this analysis used publicly available data, human subjects research approval was not sought.

## Results

During 2001–2010, a total of 256,373 confirmed Lyme disease cases were reported in the United States. The median age of patients was 42 years; 137,377 (55%) patients were male. As expected, Lyme disease cases were concentrated in the Northeast, mid-Atlantic, and upper Midwest. Standardized state-specific Lyme disease incidence rates ranged from <1 case per 100,000 person-years for 34 states to 19–73 cases per 100,000 person years for 13 high-incidence states in the Northeast, mid-Atlantic, and upper Midwest (overall median incidence 0.4 cases/100,000 person-years) (Figure).

**Figure:**
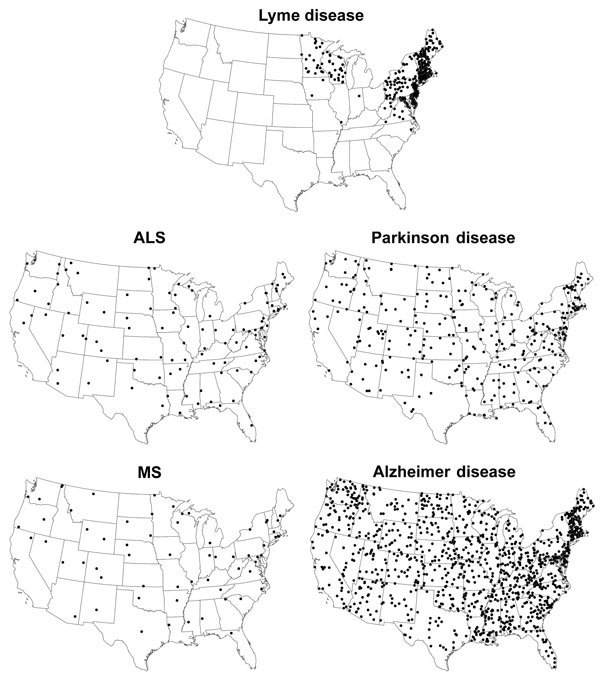
Geographic distribution of Lyme disease compared with that for deaths due to amyotrophic lateral sclerosis (ALS), Parkinson disease, multiple sclerosis (MS), and Alzheimer disease. One dot represents 1 case (Lyme disease) or 1 death (ALS, Parkinson disease, MS, and Alzheimer disease) per 100,000 person-years; dots are placed randomly within the respective states.

During the study period, 705,735 deaths were attributed to Alzheimer disease (median patient age 86 y, 29% male), 59,769 to ALS (median patient age 69 y, 54% male), 34,298 to MS (median patient age 60 y, 34% male), and 190,428 to Parkinson disease (median patient age 82 y, 58% male). Overall, disease-specific, age-adjusted median death rates ranged from a low of 1.1 deaths per 100,000 person-years for MS (range across states 0.2–2.1) to a high of 24.5 deaths per 100,000 person years for Alzheimer disease (range across states 9.6–41.2) (Figure)

All 5 diseases demonstrated positive spatial autocorrelation by the Moran’s I test: Lyme disease I index 0.49 (p<0.0001), Alzheimer disease I index 0.21 (p = 0.01), ALS I index 0.25 (p = 0.003), MS I index 0.56 (p<0.0001), and Parkinson disease I index 0.21 (p = 0.001). Nevertheless, Lyme disease incidence per state was not correlated with rates of death due to ALS (*r_s_* = 0.19, p = 0.19), MS (*r_s_* = 0.20, p = 0.17), or Parkinson disease (*r_s_* = −0.14, p = 0.34). An inverse correlation was detected between Lyme disease and Alzheimer disease (*r_s_* = −0.35, p = 0.01). Findings were similar when evaluated with Pearson correlation coefficients, when subanalysis by sex was performed, and when analysis was limited to states with >1 Lyme disease case per 100,000 person-years (data not shown).

## Discussion

As with other vectorborne diseases, Lyme disease is highly focal in its geographic distribution. If Lyme disease was etiologically linked to Alzheimer disease, ALS, MS, or Parkinson disease, rates of death attributed to these diseases would be expected to correlate geographically with the geographic incidence of Lyme disease. Our findings show, on a coarse geographic scale, no correlation between Lyme disease and these neurodegenerative conditions. The inverse correlation between the rates of Lyme disease and death from Alzheimer disease is in line with findings from a previous report (*21*); although this finding is of unclear significance, it supports a lack of positive correlation between these conditions.

Positive spatial clustering was demonstrated for each disease. This finding indicates that for each disease, states with high rates are nearer than would be randomly expected to other states that also have high rates of the disease. However, we showed that the clustering of rates of death from the 4 neurodegenerative conditions does not correlate with state-specific Lyme disease incidence, indicating that underlying processes that contribute to geographic clustering of neurodegenerative conditions are unrelated to Lyme disease. Each of the diseases, including Lyme disease, has a unique and nonrandom geographic distribution; the distributions of the neurodegenerative disorders do not mirror that of Lyme disease.

As for ALS and Parkinson disease, Lyme disease patients are more commonly male; MS and Alzheimer disease patients are more commonly female (*3*,*22*–*25*). It is conceivable that variations between states in sex distribution of the population could contribute to the lack of observed correlation between Lyme disease and the 4 neurodegenerative disorders. However, even when we examined sex-specific, age-adjusted rates by state, no correlation was identified between the geographic distribution of Lyme disease and any of the 4 neurodegenerative disorders. This finding suggests that differences in the distribution of the sexes by state did not affect the lack of correlation that we observed.

Our study had several limitations. First, our analysis was based on data from death certificates, which are limited by inconsistent practices in the completion of cause-of-death statements (*26*). Underlying cause of death is defined as the disease that initiated the chain of events resulting in death (*27*). Thus, our analysis did not include the prevalence of neurodegenerative disorders not resulting in death, and could therefore underestimate the incidence of these disorders. Nevertheless, this potential underestimation is unlikely to differentially affect the age-adjusted distribution of the deaths by state, and the death rates should proportionally reflect the prevalence of specific neurodegenerative disorders across states. Second, this cross-sectional ecologic study cannot be used to determine person-level causality. Third, the study did not account for persons who may have acquired Lyme disease in 1 state and subsequently moved to another part of the country before receiving a diagnosis of and dying from a neurodegenerative disorder. In such instances, state classification would be misclassified because of the latent period between exposure and development of disease. This direction of misclassification would reduce any observed correlation between neurodegenerative disorders and Lyme disease. Last, in historically low-incidence areas where Lyme disease has since spread, any potential time lag between infection and development of a neurodegenerative disorder would reduce the observed correlation in these areas. A limitation of the dataset was that we could not assess associations for the time from infection with Lyme disease to development of a neurodegenerative disorder.

In conclusion, although associations between Lyme disease and Alzheimer disease, ALS, MS, and Parkinson disease have been proposed by writers of speculative websites and articles, supportive evidence for such an association is lacking. The absence of a positive correlation in the geographic distributions of these conditions provides further evidence against an association between Lyme disease and deaths from these 4 neurodegenerative conditions.

## References

[1] Wormser GP, Dattwyler RJ, Shapiro ED, Halperin JJ, Steere AC, Klempner MS, The clinical assessment, treatment, and prevention of Lyme disease, human granulocytic anaplasmosis, and babesiosis: clinical practice guidelines by the Infectious Diseases Society of America. Clin Infect Dis. 2006;43:1089–134. 10.1086/508667 17029130

[2] Centers for Disease Control and Prevention. Summary of notifiable diseases—United States, 2010. MMWR Morb Mortal Wkly Rep. 2012;59:1–111 . 22647710

[3] Hinckley AF, Connally NP, Meek JI, Johnson BJ, Kemperman MM, Feldman KA, Lyme disease testing by large commercial laboratories in the United States. Clin Infect Dis. 2014;59:676–81. 10.1093/cid/ciu397 24879782 PMC4646413

[4] Bacon RM, Kugeler KJ, Mead PS; Centers for Disease Control and Prevention. Surveillance for Lyme disease—United States, 1992 to 2006. MMWR Surveill Summ. 2008;57(SS10):1–9 . 18830214

[5] Luft BJ, Steinman CR, Neimark HC, Muralidhar B, Rush T, Finkel MF, Invasion of the central nervous system by *Borrelia burgdorferi* in acute disseminated infection. JAMA. 1992;267:1364–7. 10.1001/jama.1992.03480100070033 1740859

[6] MacDonald AB. Alzheimer’s neuroborreliosis with trans-synaptic spread of infection and neurofibrillary tangles derived from intraneuronal spirochetes. Med Hypotheses. 2007;68:822–5. 10.1016/j.mehy.2006.08.043 17055667

[7] Nicolson GL. Chronic bacterial and viral infections in neurodegenerative and neurobehavioral diseases. Lab Med. 2008;39:291–9. 10.1309/96M3BWYP42L11BFU

[8] Cassarino DS, Quezado MM, Ghatak NR, Duray PH. Lyme-associated parkinsonism. Arch Pathol Lab Med. 2003;127:1204–6 . 12946221 10.5858/2003-127-1204-LPANCS

[9] Hemmer B, Glocker FX, Kaiser R, Lucking CH. Generalised motor neuron disease as an unusual manifestation of *Borrelia burgdorferi* infection. J Neurol Neurosurg Psychiatry. 1997;63:257–8. 10.1136/jnnp.63.2.257 9285472 PMC2169663

[10] Miklossy J, Khalili K, Gern L, Ericson RL, Darekar P, Bolle L, *Borrelia burgdorferi* persists in the brain in chronic Lyme neuroborreliosis and may be associated with Alzheimer’s disease. J Alzheimers Dis. 2004;6:639–49 . 15665404 10.3233/jad-2004-6608

[11] Miklossy J. Alzheimer’s disease—a neurospirochetosis. J Neuroinflammation. 2011;8:90. 10.1186/1742-2094-8-90 21816039 PMC3171359

[12] Halperin JJ. Nervous system Lyme disease: is there a controversy? Semin Neurol. 2011;31:317–24. 10.1055/s-0031-1287652 21964848

[13] Coyle PK, Krupp LB, Doscher C. Significance of reactive Lyme serology in multiple sclerosis. Ann Neurol. 1993;34:745–7. 10.1002/ana.410340521 8239571

[14] Pappolla MA, Omar R, Saran B, Andorn A, Suarez M, Pavia C, Concurrent neuroborreliosis and Alzheimer’s disease: analysis of the evidence. Hum Pathol. 1989;20:753–7. 10.1016/0046-8177(89)90068-3 2744748

[15] Marques AR, Weir SC, Fahle GA, Fischer SH. Lack of evidence of *Borrelia* involvement in Alzheimer’s disease. J Infect Dis. 2000;182:1006–7. 10.1086/315792 10950810

[16] The ALS Untangled Group. ALS untangled no.17: when ALS is Lyme. Amyotroph Lateral Scler. 2012;13:487–91. 10.3109/17482968.2012.717796 22873562

[17] Gutacker M, Valsangiacomo C, Balmelli T, Bernasconi MV, Bouras C, Piffaretti JC. Arguments against the involvement of *Borrelia burgdorferi* sensu lato in Alzheimer’s disease. Res Microbiol. 1998;149:31–7. 10.1016/S0923-2508(97)83621-2 9766207

[18] Halperin JJ, Luft BJ, Anand AK, Roque CT, Alvarez O, Volkman DJ, Lyme neuroborreliosis: central nervous system manifestations. Neurology. 1989;39:753–9. 10.1212/WNL.39.6.753 2542840

[19] Schmutzhard E, Pohl P, Stanek G. *Borrelia burgdorferi* antibodies in patients with relapsing/remitting form and chronic progressive form of multiple sclerosis. J Neurol Neurosurg Psychiatry. 1988;51:1215–8. 10.1136/jnnp.51.9.1215 3225603 PMC1033030

[20] Qureshi M, Bedlack RS, Cudkowicz ME. Lyme disease serology in amyotrophic lateral sclerosis. Muscle Nerve. 2009; 40: 626–8. 10.1002/mus.21438 19697382

[21] O’Day DH, Catalano A. A lack of correlation between the incidence of Lyme disease and deaths due to Alzheimer's disease. J Alzheimers Dis. 2014;42:115–8. 24840565 10.3233/JAD-140552

[22] Walling AD. Amyotrophic lateral sclerosis: Lou Gehrig’s disease. Am Fam Physician. 1999;59:1489–96 . 10193591

[23] Rao SS, Hofmann LA, Shakil A. Parkinson’s disease: diagnosis and treatment. Am Fam Physician. 2006;74:2046–54 . 17186710

[24] Calabresi PA. Diagnosis and management of multiple sclerosis. Am Fam Physician. 2004;70:1935–44 . 15571060

[25] Hebert LE, Scherr PA, McCann JJ, Beckett LA, Evans DA. Is the risk of developing Alzheimer’s disease greater for women than for men? Am J Epidemiol. 2001;153:132–6 . 10.1093/aje/153.2.132 11159157

[26] Smith Sehdev AE, Hutchins GM. Problems with proper completion and accuracy of the cause-of-death statement. Arch Intern Med. 2001;161:277–84 . 10.1001/archinte.161.2.277 11176744

[27] Centers for Disease Control and Prevention. Instructions for completing the cause-of-death section of the death certificate [cited 2014 Jun 13]. http://www.cdc.gov/nchs/data/dvs/blue_form.pdf

